# High-Throughput Screening for Spermatogenesis Candidate Genes in the AZFc Region of the Y Chromosome by Multiplex Real Time PCR Followed by High Resolution Melting Analysis

**DOI:** 10.1371/journal.pone.0097227

**Published:** 2014-05-14

**Authors:** Evguenia Alechine, Daniel Corach

**Affiliations:** Servicio de Huellas Digitales Genéticas, Facultad de Farmacia y Bioquímica, Universidad de Buenos Aires, Buenos Aires, Argentina; CCR, National Cancer Institute, NIH, United States of America

## Abstract

Microdeletions in the AZF region of the Y chromosome are among the most frequent genetic causes of male infertility, although the specific role of the genes located in this region is not fully understood. AZFa and AZFb deletions impair spermatogenesis since no spermatozoa are found in the testis. Deletions of the AZFc region, despite being the most frequent in azoospermic patients, do not correlate with spermatogenic failure. Therefore, the aim of this work was to develop a screening method to ascertain the presence of the main spermatogenesis candidate genes located in the AZFc region in the light of the identification of those responsible for spermatogenic failure. *DAZ, CDY, BPY2, PRY, GOLGA2LY* and *CSGP4LY* genes were selected on the basis of their location in the AZFc region, testis-only expression, and confirmed or predicted protein codification. *AMEL* and *SRY* were used as amplification controls. The identification of Real Time PCR products was performed by High Resolution Melting analysis with SYTO 9 as intercalating dye. The herein described method allows a rapid, simple, low-cost, high-throughput screening for deletions of the main AZFc genes in patients with spermatogenic failure. This provides a strategy that would accelerate the identification of spermatogenesis candidate genes in larger populations of patients with non-obstructive idiopathic azoospermia.

## Introduction

Infertility affects about 15% of the couples trying to conceive. In half of the cases, the underlying cause can be partially or totally attributed to the male partner, and 10–15% of the cases could be due to genetic abnormalities [1]. For many years, microdeletions in the euchromatic region of the Y chromosome have been linked to spermatogenic failure, as cytogenetic studies associated a deletion on its long arm (Yq) to azoospermia [2]. The azoospermia factor region (AZF) and its sub-regions AZFa, AZFb and AZFc are the main targets for molecular diagnosis [3], [4]. These AZF loci are routinely screened for possible microdeletions using sequence tagged sites (STSs), as recommended by the European Academy of Andrology (EAA) [5]. This diagnostic testing of Yq microdeletions is generally performed by PCR amplification of selected STSs on the Y chromosome. The precise sequencing and mapping of the male-specific region of the Y chromosome clarified that many STSs used in initial screening were polymorphic, ambiguous, heterogeneous and unreliable, prone to produce technical problems and false results [6], [7]. The use of gene-specific primers flanking specific locations enables to obtain information on the potential mechanisms, and the extension of the deletions. These studies also highlighted the improved value of including gene-specific markers in the minimal set of markers for the analysis [8].

On the other hand, complete deletions of specific AZF regions have been associated with different spermatogenic scenarios such as: AZFa to Sertoli cell-only syndrome, AZFb/AZFbc to spermatogenic arrest with interruption in meiosis I, and AZFc to hypospermatogenesis leading to severe oligozoospermia and azoospermia. Interestingly, microdeletions of either the entire AZFa or AZFb regions heralds a poor prognosis for sperm retrieval from testicular sperm extraction (TESE) or testicular biopsy, whereas half of the men with complete AZFc deletion have successful sperm retrieval [1]. Nonetheless, the AZFc region is the most frequently deleted in patients with non-obstructive azoospermia or severe oligozoospermia, attaining about 70% of all the cases. From previous observations, neither it is possible to infer the phenotype from the classic genetic testing for the AZFc region nor to identify the genes essential for normal spermatogenesis that this sub-region harbors [5]. Hence, in order to provide a strategy that would accelerate the identification of spermatogenesis candidate genes, our goal was to develop a method that would allow the screening of the main AZFc genes in large number of patients with non-obstructive idiopathic azoospermia or severe oligozoospermia.

In terms of gene content, the AZFc region contains 11 families of transcription units expressed only in the testis. Five of them code for proteins, four are pseudogenes, and two have open reading frames [9]. Namely, the *deleted in azoospermia* (*DAZ*) gene family is expressed in spermatogonia and encodes a RNA-binding protein important for spermatogenesis. Four copies of this gene (*DAZ1, DAZ2, DAZ3* and *DAZ4*) are located in AZFc [9], [10], [11]. The *chromodomain protein Y* (*CDY*) gene family encodes a protein containing a chromodomain and a histone acetyltransferase catalytic domain located in the nucleus of late spermatids where histone hyperacetylation takes place. The human Y chromosome has two identical copies of this gene within the AZFc region (*CDY1A* and *CDY1B*) and a pair of closely related genes in the palindrome P5 (*CDY2A* and *CDY2B*) [6], [12]. The *testis-specific basic protein Y 2* (*BPY2*) gene is expressed specifically in testis and its protein product is involved in male germ cell development and male infertility. Three nearly identical copies of this gene exist on Y chromosome [13]. The *PTPBL-related gene on Y* (*PRY*) is expressed specifically in testis and encodes a protein with similarity to protein tyrosine phosphatase, non-receptor type 13. Two nearly identical copies of this gene exist on the Y chromosome [14]. *Golgi autoantigen, golgi subfamily a, 2-like, Y-linked* (*GOLGA2LY*) and *chondroitin sulfate proteoglycan 4 pseudogene 1, Y-linked* (*CSPG4P1Y*) families of transcription units have significant open reading frames, are expressed only in testis and have predicted protein products. Both have 2 copies located in P1 palindrome [9].

The previous are the main protein-coding genes located in the AZFc region that are potentially related to spermatogenic failure, although the specific contribution to spermatogenesis of each of the individual genes is still not fully understood. Aiming to clarify the implication of the main candidate genes in spermatogenic failure, we selected these genes for a deeper understanding of how Y chromosome microdeletions, and specifically the deletion of AZFc-located genes, affect normal spermatogenesis. Herein we designed a one-step multiplex PCR reaction for the identification of the complete absence of six AZFc genes. The developed method allows a rapid, simple, low-cost, high-throughput screening for deletions of the main AZFc genes in patients with non-obstructive idiopathic azoospermia.

## Results

### Design of the multiplex Real Time PCR/HRM method

As a first approach in order to develop a multiplex Real Time PCR (rtPCR) system with High Resolution Melting analysis (HRM) for AZFc gene screening we designed primers for the specific amplification of all the copies of six AZFc genes. The following genes were selected on the basis of their location in the AZFc region of the Y chromosome, testis-only expression and confirmed or predicted protein codification: *DAZ, CDY, BPY2, PRY, GOLGA2LY* and *CSGP4LY*. Furthermore, *SRY* was selected as internal amplification control, and *AMELxy* as internal and specificity control. These primers were combined to establish one single 8-plex reaction. High peak discrimination was achieved by selecting amplification products with different predicted melting temperature (Tm) values for each peak.

**Figure 1:**
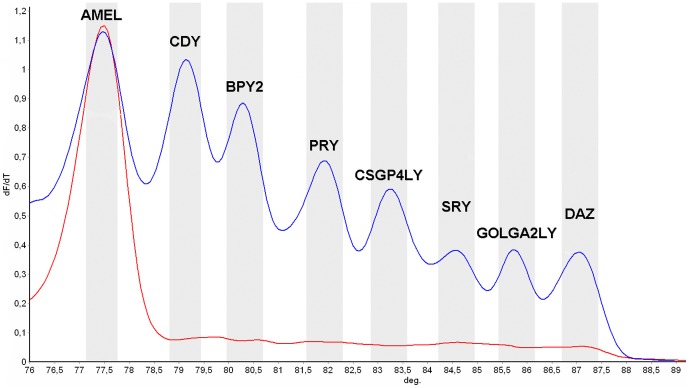
High Resolution Melting derivative (dF/dT) profile for male and female samples. The figure shows each of the peaks for every amplified gene in the male sample (blue) and only AMELxy peak in the female sample (red).

As a first step, we amplified normal undeleted male samples as well as female samples attaining high amplification levels for all the selected genes in male samples, while no amplification (except for *AMELxy*) was observed in female samples (Figure 1). This optimal condition was achieved by increasing the annealing temperature of the PCR to 62°C. In order to confirm the specificity of the amplification, all PCR products were sequenced and aligned to the GeneBanks reference sequence, matching the genes of interest.

### Performance of the method to detect specific gene deletions

**Figure 2:**
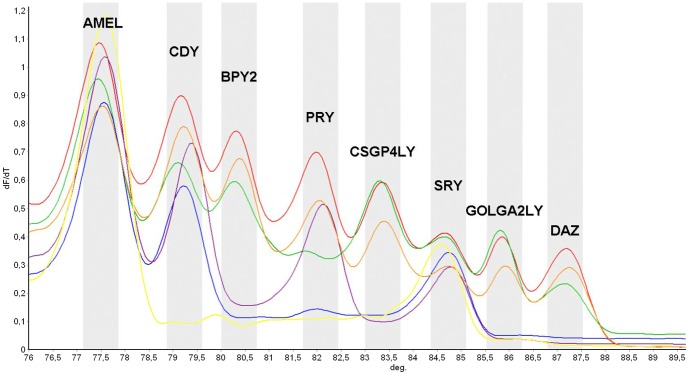
High Resolution Melting derivative (dF/dT) profile for deleted and undeleted samples. The figure shows AZFa-deleted (orange) and undeleted (red) samples with no gene deletions, AZFb-deleted sample with lack of amplification only of PRY gene (green), AZFc-deleted with no amplification of BPY2, DAZ, GOLGA2LY and CSGP4LY genes (violet), AZFbc-deleted with no amplification of PRY, BPY2, DAZ, GOLGA2LY and CSGP4LY genes (blue), and AZFabc with the sole amplification of AMEL and SRY genes (yellow), as evidenced by the corresponding missing peaks.

We tested control samples with AZFa, AZFb, AZFbc and AZFc deletions in order to characterize the ability of the system to identify the specific deletion of the genes located in these regions. Deleted samples were previously analyzed by a modified for Real Time EAA protocol in order to detect classical AZF microdeletions [15] and then undeleted samples were further analyzed for partial AZFc microdeletions by means of STSs analysis, including sY142, sY1191, sY1197, sY1201, sY1206, sY1261 and sY1291 markers [9], [16]. As expected, inferred from the GenBanks Y chromosome reference sequence, the AZFa-deleted sample evidenced no deletion of the analyzed genes, conversely to AZFb-deleted that showed lack of amplification for *PRY* gene, AZFc-deleted no amplification of *BPY2, DAZ, GOLGA2LY* and *CSGP4LY* genes, and AZFbc-deleted no amplification of *PRY, BPY2, DAZ, GOLGA2LY* and *CSGP4LY* genes. Only those samples with complete AZFabc deletion were negative for all the genes, while positive for AMEL and SRY amplification controls (Figure 2). When HRM data analysis was performed with Rotor Gene software, which allows the automatic identification of the peaks in the derivative (dF/dT) plot, the assignment of the sample to a specific genotype was enabled.

**Figure 3:**
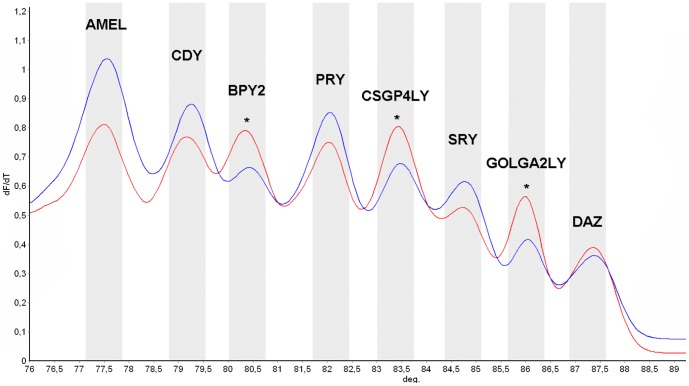
High Resolution Melting derivative (dF/dT) profile for undeleted and b2/b3 deleted samples. The figure shows each of the peaks for every amplified gene for undeleted (red) and b2/b3 deleted (blue) samples. In BPY2, GOLGA2LY and CSGP4LY genes, an inversion of the peak high ration is observed (noted by asterisks) as a consequence of the incomplete deletion of these genes.

Furthermore, we analyzed samples typified with Y chromosome STSs markers routinely employed to identify gr/gr, b1/b3 and b2/b3 partial AZFc microdeletions [16]. As expected, no peak loss was observed as a consequence of the remaining gene copies. Although no diagnosis of partial AZFc deletions could be performed by this technique, a variation of peak height was observed in partially deleted samples, most likely due to the decrease of the remaining gene copy number. For example, in b2/b3 deleted samples an inversion of peak height was observed for *BPY2*, *CSGP4LY* and *GOLGA2LY* genes, which are located within the deleted region (Figure 3). The same effect was observed in samples with an AZFb deletion, where a decrease in peak height for CDY, BPY2 and DAZ was observed as a consequence of gene copy reduction.

### Performance of three different intercalating dyes for multiplex HRM

As the amplification was tested with three intercalating dyes, namely SYBR Green I, EvaGreen and SYTO 9, it was possible to compare their performance in multiplex amplification and HRM peak discrimination. At first, the 8-plex reaction was set up with SYTO 9 achieving high sensitivity, optimal melting peak resolution and correlation between predicted and real Tm. When the same reaction was reproduced with SYBR Green I, sensitivity was compromised obtaining about 30% less fluorescence than with SYTO 9 and peak loss was observed (Figure S1; green). Likewise, when the reaction was performed with EvaGreen, a shift in the predicted melting peaks towards higher temperatures was observed (Figure S1; blue). Moreover, both tested commercial kits are more expensive and require more DNA and primer input, in contrast to SYTO 9 that allowed a fast setup of the multiplex reaction with minimal costs. Therefore, we designed a standard homemade PCR master mix with a universal intercalating dye as SYTO 9 and regular desalted primers, plus DNA and primer concentrations used have been reduced to a minimum without losing sensitivity.

### Robustness and sensitivity

To test the performance of the multiplex for robustness regarding peak strength and separability, we analyzed 10 different genomic DNA samples from males without AZFc deletions. Regardless the origin of the sample, the melting peaks from every sample were present and could be clearly distinguished. Furthermore, robustness was tested by a blind assay performed by a different co-worker in which 5 undeleted controls and three samples from AZFc deleted patients (b2/b4) were analyzed. In agreement with the corresponding deleted region, the AZFc deleted samples were correctly identified as missing *BPY2, DAZ, GOLGA2LY* and *CSGP4LY* genes (data not shown).

The sensitivity of the system was evaluated by using genomic DNA from an undeleted control. We performed the PCR reaction with two-fold dilutions of the control sample ranging 20–0.312 ng/µL. The individual melting peaks could be accurately identified at concentrations bellow 10 ng/µL, despite that no linear correlation between DNA concentration and signal strength was evidenced. It is worth to notice that despite the complexity of the multiplex reaction, all samples extracted by semi-automated methods (usually ranging from 1 to 10 ng/µL) could be easily analyzed, even avoiding previous quantification.

### Portability of the method to a 96-well block based system

**Figure 4:**
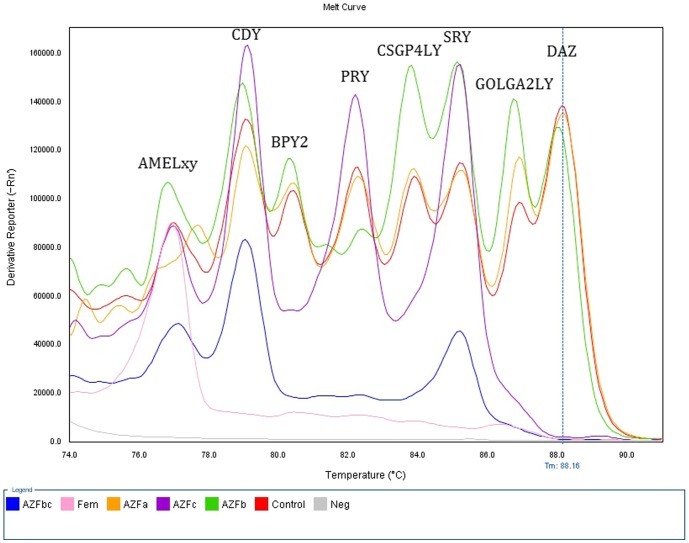
High Resolution Melting derivative (dF/dT) profile for undeleted and deleted samples obtained with StepOne Plus equipment. The figure shows AZFa-deleted (orange) and undeleted (red) samples with no deletions, female sample with only AMELxy peak presence (pink), AZFb-deleted sample with lack of amplification only of PRY gene (green), AZFc-deleted with no amplification of BPY2, DAZ, GOLGA2LY and CSGP4LY genes (violet), and AZFbc-deleted with no amplification of PRY, BPY2, DAZ, GOLGA2LY and CSGP4LY genes (blue), as evidenced by the corresponding missing peaks.

As the multiplex system was established on a rotary with moving hot air-based heating system Real Time PCR equipment (Rotor Gene 6000), it was worthy to check portability of the method to a different equipment. Hence, the reaction was also tested on a 96-well block based system (StepOne Plus, Life Technologies, USA). The latest is based on a heating block with LED based 4-color optical detection. The multiplex reaction was reproduced exactly as it was set up for Rotor Gene with the unique modification of the melting curve stage following the manufactureŕs indications. We also obtained an optimal peak separability, although the analysis with StepOne Plus software was only qualitative due to the limitation of the software to assign multiple melting peaks, hampering the simultaneous analysis of all the genes in a high number of samples (Figure 4).

### Clinical validation of the method

The validation of the method was performed by analyzing 140 samples from patients consulting for infertility (screening group), and 90 samples from azoospermic patients that were candidates for testicular biopsy (azoospermic group). In the screening group, only one patient with a complete AZFc or b2/b4 deletion showed the absence of *DAZ*, *GOLGA2LY*, *CSGP4LY* and *BPY2* genes. In the azoospermic group, those with complete deletions of either or both AZFb or AZFc regions were lacking the genes located in these regions, namely *DAZ*, *GOLGA2LY*, *CSGP4LY* and *BPY2* in AFZc, plus *PRY* in AZFb. One sample with a complete AZFabc deletion showed the absence of all the analyzed genes. All the samples with gene deletions were azoospermic or severely oligozoospermic, and none of the samples with sperm count above 5×10^6^/mL evidenced any gene deletion.

## Discussion

The characterization of spermatogenesis candidate genes may lead to improvements in the diagnosis and treatment of male patients suffering from infertility. Several methods for the identification of the genes involved in Y chromosome microdeletions have been developed, although the majority did not allowed large population screening. Herein we propose an 8-plex Real Time PCR method with High Resolution Melting analysis for the main AZFc candidate genes. The reaction was designed for the specific amplification of every copy of six AZFc genes. The genes were selected on the basis of their location in the AZFc region of the Y chromosome, testis only expression, and confirmed or predicted protein codification. Primer sequences and multiplex primer combinations were selected to achieve high separation of melting peaks improved by the use of SYTO 9 intercalating dye attaining the identification of gene presence without downstream post PCR analysis.

Several detection chemistries are available for melting curve analysis by Real Time PCR. Oligonucleotide probes or modified/labeled primers are commercially available; nevertheless non-sequence specific DNA dyes are more cost effective and equally applicable. Among the latest, SYBR Green I has been widely used although it suffers from several drawbacks as PCR inhibition and dye redistribution. Other alternatives are SYTO or EvaGreen dyes, both can be used at saturating concentrations without affecting PCR efficiency and enable higher resolution melting curve analysis [17]. From our results we evidenced that in contrast to SYBR Green I; EvaGreen and SYTO 9 showed higher sensitivity and broader peak separability. SYBR Green I failed in amplifying all the products, in agreement with previous studies that showed that SYRB Green I selectively detect particular amplicons in melting curve analysis [18]. In contrast, SYTO 9 correlated better between predicted and real melting peaks, meanwhile EvaGreen showed a switch of melting peaks towards higher temperatures, probably due to stabilizing interactions of the dye with the DNA. From our experience, the prediction of the ampliconś melting temperatures was accurately achieved by means of Oligo Calculator v3.26 software. Additionally, as high reaction efficiencies and signal strength are achievable with these dyes, DNA and primer concentrations have been reduced to a minimum without losing sensitivity. The development of a standard homemade PCR with a universal intercalating dye as SYTO 9 and regular desalted primers allowed a fast setup of the multiplex reaction.

Although the advantage of the Rotor Gene-based data analysis was the automatic identification of individual melting peaks in a multiplex HRM derivative (dF/dT) profile, the reaction was successfully reproduced in a heating block-based Real Time PCR system achieving an optimal detection of each melting peak. It should be taken into account that some differences in signal strength are likely due to differential absorption wavelengths of the instruments.

In order to guarantee the result of the analysis, *AMEL* gene amplification was included to detect the presence of both X and Y chromosome *AMEL* genes. *SRY* gene was amplified in order to identify the presence of the Y chromosome and as an internal control of the reaction. Due to the presence of Y chromosome homologue genes located on the X and autosomal chromosomes, the importance of *AMELxy* amplification in addition to female control analysis rely on discarding unspecific amplification of autosomal or X homologues in Y chromosome deleted samples. The designed protocol has a high level of specificity for the AZFc genes in order to ensure that the presence of a peak is only due to the presence of the gene of interest.

In addition, when amplification products were checked by 3% agarose gel electrophoresis to verify product size, this technique allowed the identification of several bands, although some of the products were not resolved due to similar molecular weight. Despite being a low cost technique, agarose gel electrophoresis of multiplex PCR products is not suitable for distinguishing between products of similar size; instead their melting temperatures can successfully differentiate the same products.

Interestingly, in partial AZFc deleted samples, although no peak loss was detected, inversion of peak height was observed in those genes involved in the deletion. Even though it was not the aim of the developed protocol it is also possible to qualitatively characterize samples with partial AZFc deletion by analyzing melting peak height within the same sample.

Not less important is the DNA input used to perform the PCR reaction. Although DNA quantification was performed, the herein described method is useful within a DNA input ranging from 1 to 10 ng (data not shown). This amount is effortless achieved using the standardized DNA extraction method herein described, so DNA quantification step could be avoided.

The still unclear genotype–phenotype relationship reflects the current knowledge about the AZFc region. AZF regions include various genes which function is still unknown. Y chromosome microdeletions are generally assessed for diagnostic purposes using a set of anonymous STSs that are frequently not specific for the genes located in this region. Within the markers included in the screening protocol suggested by the European Academy of Andrology, the only gene-specific STS is located within the *DAZ* gene [5]. Therefore, the general trend should be to study the Y chromosome using gene-specific markers (complementary to classical STSs markers). In this way, the diagnosis will be more accurate, and the study of a greater number of infertile patients with this protocol will probably allow us to better understand the biology of spermatogenesis and to clarify the genotype–phenotype relationships. In addition, excluding *DAZ*, *RBMY*, *USP9Y* and *DBY* genes, little is known about the other genes mapping the AZF regions. Only future studies will clarify if these are involved in human spermatogenesis and male infertility, and if they should be included in the screening protocol. Our protocol includes genes that have not yet been widely studied as *PRY*, *GOLGA2LY* and *CSGP4LY* genes. It is worthy to mention that many of the findings made on this matter couldńt have been possible with the current proposed commercial kits for the screening of Y microdeletions and additional approaches should be included [19], [20], [21].

A similar and widely used approach for identification of AZFc genes is the gene dosage by quantitative PCR [16] or capillary electrophoresis [22]. Even though gene dosage analysis permits the quantification of specific genes, and therefore also the identification of complete absence of them, it should be performed in a simplex reaction. The limitation of quantifying a single gene relays on the unspecific bound of intercalating dyes to all amplification products, therefore not distinguishing between different genes. This limitation can be overcome by labeling each primer pair with different fluorophores, increasing significantly the cost of the analysis. On the contrary, the analysis of the HRM profile of a multiplex reaction makes it possible to detect the lack of amplification of individual genes in a background of other positively amplifying genes. Furthermore it has been shown that gene copy number reduction does not correlate with the observed phenotype; high similarity within gene copies make it likely to replace in function those genes lost by deletion. Therefore we suggest that the identification of genes involved in spermatogenic failure should be performed to detect complete absence of the genes.

On the other hand, capillary electrophoresis has been also widely used specially for relative quantification of *DAZ* and *CDY* genes. In this case, an autosomal homologue is amplified in parallel to the gene of interest and the peak height ratio between the autosomal and Y gene is analyzed. This approach, although useful, has two main disadvantages: the inherent need for the amplification of a homologue to each gene of interest and the higher cost of labeled primers and capillary electrophoresis equipments and supplies. With this in mind, both gene dosage by qPCR and capillary electrophoresis are less suitable techniques for large population screening due to higher costs and lower throughput.

Although this newly developed method does not aim to detect gene conversion on the AZF region of the Y chromosome, it is important to notice that this phenomena may lead to translocation or modification of the copy number of the genes located in this region [23]. Taking into account that is still not clear which of the copies of these genes are involved in spermatogenesis, we underscore the importance of analyzing the presence/absence of all the copies. In those samples where at least one of the gene copies is still remaining, as the melting peak is observed, further analysis of sequence family variants (SFVs) of these genes is suggested to identify which of the missing copies could be involved in the patients' phenotype. In order to perform this analysis there are several protocols available aiming to identify single nucleotide polymorphisms (SNPs) in *DAZ* and *CDY* genes that are able to differentiate between copies of these genes [24], [25].

As previously published by our group, it is possible to employ the HRM analysis tool with intercalating dyes not only for simplex but also for multiplex amplifications. In a previous work, we developed a technique to simultaneously detect from three to five SNPs by means of one single amplification [26]. With this background, the analysis of several genes by multiplex amplification with intercalating dyes was an approachable goal. To our knowledge, this is the first work to analyze six spermatogenesis candidate genes in a multiplex approach. By means of the herein described protocol it is possible to analyze six genes in up to 72 samples in less than 2 hours, making it the ideal tool for screening large number of patients with idiopathic infertility. The developed method uses common reagents, available in any molecular biology laboratory, instead of labeled commercial kits that not always provide with additional information to the assay. This approach is useful for screening patients with non-obstructive azoospermia or severe oligozoospermia, additionally to classical AZF and partial AZFc deletions, and to being further correlated with testicular biopsies findings.

We consider our work to be of high relevance to researchers focused either on molecular basis or clinical aspects, mainly diagnosis, of spermatogenic failure. Additionally, the approach described in our work, although targeted to spermatogenesis genes, can be applied to other research fields were gene screening is relevant.

## Materials and Methods

### DNA samples

We used DNA from blood samples or cell lines to set up the reaction. Normal undeleted and AFZc-deleted (b2/b4, b2/b3 and gr/gr) male samples were selected from routine AZF screening. Control DNA for P5/proximal P1 or AZFb-deleted (NA18332), P5/distalP1 or AZFbc-deleted (NA18333), and b1/b3-deleted (NA20434) Y chromosomes was purchased from Coriell Cell Repository (New Jersey, USA). Human male DNA from AZFa-deleted patient (WHT2996) was a courtesy of Dr. David C. Page from the Whitehead Institute for Biomedical Research (Cambridge, USA) [27]. As a specificity control, female samples have been amplified in parallel. Samples used for the clinical validation of the method were obtained from patients concurring either to the Laboratory of Male Infertility or to the Urology Department, both from the Hospital de Clínicas “Jose de San Martín”. All sample donors signed an informed consent. The ethics committee of the School of Pharmacy and Biochemistry of the University of Buenos Aires has approved the present study; nevertheless all the samples were treated anonymously. All investigation has been conducted according to the principles of the Declaration of Helsinki.

### DNA extraction and quantification

DNA from blood samples spotted onto Whatman 3MM paper was extracted by means of a semi-automated platform (Maxwell, Promega, USA) using manufacturer's reagents and protocols. Quantification of extracted DNA was performed by means of Real Time PCR using a commercial kit (Plexor HY, Promega, USA) and a Rotor Gene 6000 Real Time PCR equipment (Corbett Research, Australia).

### Primer design

**Figure 5:**
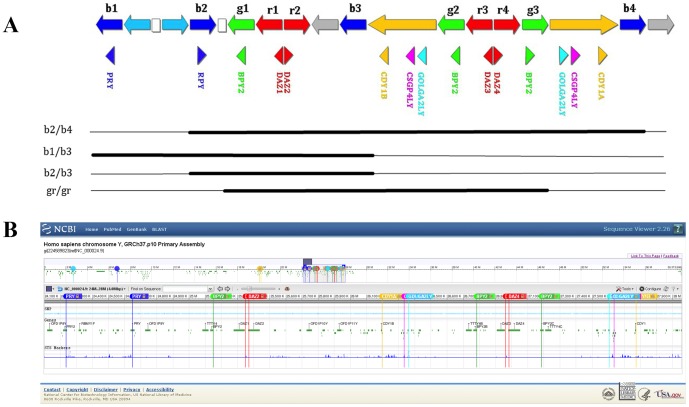
Scheme of the AZFc region with amplicon and gene copy locations, (a). The figure shows the organization of the amplicon sequences on the AZFc region of the Y chromosome indicated with arrows and selected genes indicated with triangles. Complete and partial AZFc deletions are shown below. Ampliconic sequenceś nomenclature is according to Kuroda-Kawaguchi et al (2001). Location of the amplification products on the reference Y chromosome, (b). The location is based on Homo sapiens chromosome Y, GRCh37.p10 Primary Assembly (http://tinyurl.com/d7s5ztr).

NCBI Y chromosome reference sequence was searched for the genes of interest. Optimal primers were designed using Primer3 software (http://frodo.-wi.mit.edu/primer3) [28]. The predicted amplification products were checked using UCSC Genome Browser online software (http://genome.ucsc.edu/cgi-bin/hgGateway) and primers amplifying all the copies of the gene of interest were selected. In order to obtain broader divergence between melting peaks, ampliconś melting temperature (Tm) was predicted using Oligo Calculator v3.26 (http://www.basic.northwestern.edu/biotools/OligoCalc.html). Location of the selected amplification fragments is shown in Figure 5a and 5b. All amplified sequences are shown along the Y chromosome using the NCBÍs Sequence Viewer 2.26 software (http://tinyurl.com/d7s5ztr).

### Multiplex PCR conditions

Multiplex amplification was tested with three intercalating dyes included in different multiplex reactions, namely SYTO 9, SYBR Green I (Life Technologies, USA) and EvaGreen (Biotium, USA). SYBR Green I was part of the Quantitec SYBR Green PCR kit (QIAGEN, Germany) and EvaGreen of Mezcla Real (Biodynamics, Argentina). Commercial kit protocols were performed as suggested by the manufacturer. Meanwhile, the herein developed protocol employing SYTO 9 as intercalating dye was performed as follows. Multiplex PCR reaction was carried out in 25 µL final volume containing 37.5 pmol SYTO 9, 2 units GoTaq DNA Polymerase (Promega, USA), 5× Colorless GoTaq Reaction Buffer (Promega, USA), 20 nmol 4 dNTPs mix (Promega, USA), primers (Integrated DNA Technologies, USA) (as indicated in Table 1) and 2 µL DNA template (with a concentration between 0.3125 and 10 ng/µL). A detailed protocol is described in Protocol S1 (see Supporting Information). Primer sequences and concentrations used in all the reactions are listed in Table 1. The reactions were performed in a Rotor Gene 6000 (Corbett Life Science, Australia) and in a StepOne Plus (Life Technologies, USA) Real Time PCR equipment. PCR cycling conditions were the following: initial denaturation step at 95°C for 1 min followed by 30 cycles of 94 °C 10 s/62 °C 30 s/72 °C 30 s. Following amplification, a High Resolution Melting (HRM) analysis was performed ranging from 74 °C to 91 °C at 0.2 °C/s for Rotor Gene 6000. When using StepOne Plus, the melting curve stage was set as follows: 95°C 15 s–60°C 1 min followed by a 0.3% continuous ramp from 74 °C to 91 °C. Likewise, the cycling profile has been modified when Hot Start polymerase was used, as with Quantitec SYBR Green PCR kit.

**Table 1:** Primers used for the multiplex PCR amplification.

Gene	Primer sequence	Primer input	Product size	Predicted Tm	Real Tm	N°Copies
	(5′-3′)	(pmol)	(bp)	(°C) *	(°C) **	detected
AMEL	*ATCAGAGCTTAAACTGGGAAGCTG*	8	106/112	76.3/76.8	77.5±0.5	1 or 2
	*CCCTGGGCTCTGTAAAGAATAGTG*	8				
CDY	*TGGAGAGGTTCAGGCACATG*	13.5	111	78	79.2±0.5	4
	*TCCAGCTCTTCACCAGGTTC*	13.5				
BPY2	*GGCAAGGTTCCTCCTTATCC*	9	115	80.4	80.4±0.5	3
	*CACTTTCGAGGTTGGAAGGA*	9				
PRY	*GCTGCCTCCTCTCATCTGTC*	13.5	114	82.4	82.0±0.5	2
	*TGGAAACTTCATCCCAGAGG*	13.5				
CSGP4LY	*AGGCTGATGTTGGAGGATTG*	9	154	82.6	83.4±0.5	2
	*TCATGCCAAGAAGACACCTG*	9				
SRY	*GCCACACACTCAAGAATGGA*	15.6	124	83	84.7±0.5	1
	*CCAATGTTACCCGATTGTCC*	15.6				
GOLGA2LY	*AGCCTTTTCCTTGTGGAGGT*	9	366	85.4	85.9±0.5	2
	*GTGGACTCTGAGCCTCTTGG*	9				
DAZ	*TCTGTGCCTGCCTCTCTGTA*	15.5	121	86	87.4±0.5	4
	*GCCTTATCCTCGGTTTTCCT*	15.5				

* Melting temperature (Tm) was predicted using Oligo Calculator v3.26.

** Melting temperature obtained with the newly developed SYTO 9 protocol and amplification in Rotor Gene 6000.

### Sequencing of the amplification products

For confirmation of the specificity of the amplification, all PCR products were cleaned up using Illustra ExoStar Kit (GE Healthcare, UK) and sequenced with both forward and reverse primers using the Big Dye Terminator Cycle Sequencing kit v3.1 (Life Technologies, USA), according to the supplier's protocol. Purification of the sequences from residual dye terminators was performed by Isopropyl alcohol 75%/Ethanol 70% precipitation. Capillary Electrophoresis of the sequences was carried out in an ABI3500 Genetic Analyzer (Life Technologies, USA). Electropherograms were visualized and edited with Sequencher v4.8 software (Gene Codes Corporation, USA). The obtained sequences were aligned with GeneBanks reference sequence.

### Analysis of classical AZF microdeletions

Classical AZF microdeletions were analyzed by the EAA protocol, modified for Real Time PCR [15]. Briefly, AZFa, AZFb, AZFc and AMEL primers were used. The protocol was adapted and setup for amplification with SYTO 9 as intercalating dye. From the primer combinations described by Kozina et al. we selected those from Setup 2 (AMELxy, sY255, sY127 and G34990), with the exception of G34990 primer that, due to unspecific amplification of a sequence located on the X chromosome, was switched for sY85.

### Analysis of partial AZFc microdeletions

Partial AZFc microdeletions were analyzed by plus/minus amplification of validated Sequence Tagged Sites (STS) markers located in the Y chromosome [9], [16]. Briefly, markers sY142, sY1197, sY1191, sY1291, sY1206, sY1201 and sY1261 have been amplified. Amplification was performed in a multiplex reaction with 12.5 µL Multiplex PCR Master Mix (QIAGEN, Germany), 5 pmol FAM-labeled M13 primer (FAM-*TGTAAAACGACGGCCAGT*), unlabeled primers (listed in Table S1) and 2 µL template DNA (ranging 1–10 ng/µL) in a total volume of 25 µL. The cycling conditions were the following: 15 minutes for Taq polymerase activation at 95°C, 30 cycles of 95°C 30 sec, 60°C 30 sec and 72°C 30 sec, and 8 cycles of 95°C 30 sec, 53°C 30 sec and 72°C 30 sec, followed by 30 minute extension at 72°C. All PCR reactions were performed in a Verity Thermal Cycler (Life Technologies, USA). PCR primerś sequences have been designed with M13 tails for product detection with M13 primers labeled with FAM, as previously described [29]. PCR productś resolution and detection was performed with a 3500 Genetic Analyzer (Life Technologies, USA) and the analysis of the results was performed with GeneMapper ID-X v1.2 software (Life Technologies, USA).

## Supporting Information

Figure S1 **High Resolution Melting derivative (dF/dT) profile testing the performance of SYTO 9, SYBR Green I and EvaGreen.** The figure shows the bin locations according to standard amplification with SYTO 9 of an undeleted sample (red). EvaGreen shows optimal amplification of all the genes but with approximately 1°C shift in melting temperature (blue). SYBR Green I shows lack of detection of BPY2 genes, shift in melting temperature and imbalance in melting peak height within all the genes (green). The comparison was performed in standard amplification conditions and 10 ng of DNA input.
(TIF)

Protocol S1 **Detailed SYTO 9 multiplex reaction protocol.**
(DOC)

Table S1 **Primers used for Y-STS amplification.**
(DOC)
