# Supplementary figures and images for: High-Throughput Screening for Spermatogenesis Candidate Genes in the AZFc Region of the Y Chromosome by Multiplex Real Time PCR Followed by High Resolution Melting Analysis
Source: PLoS One. 2014 May 14;9(5):e97227. doi: 10.1371/journal.pone.0097227 (PMC4020812; doi:10.1371/journal.pone.0097227)

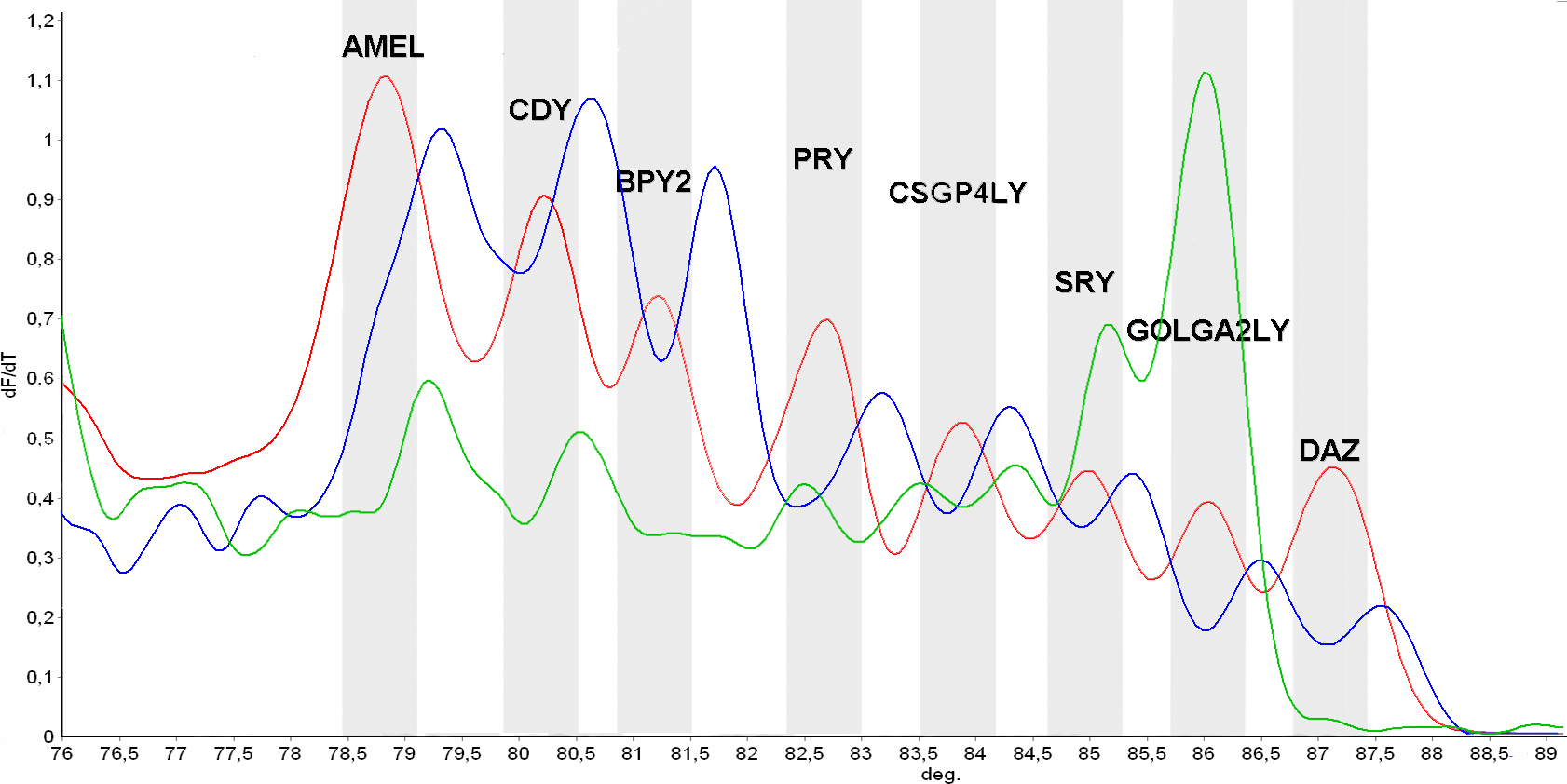

Supplement: Figure S1 — High Resolution Melting derivative (dF/dT) profile testing the performance of SYTO 9, SYBR Green I and EvaGreen. The figure shows the bin locations according to standard amplification with SYTO 9 of an undeleted sample (red). EvaGreen shows optimal amplification of all the genes but with approximately 1°C shift in melting temperature (blue). SYBR Green I shows lack of detection of BPY2 genes, shift in melting temperature and imbalance in melting peak height within all the genes (green). The comparison was performed in standard amplification conditions and 10 ng of DNA input. (TIF) [file pone.0097227.s001.tif]
