# Supplementary material for: High-Throughput Screening for Spermatogenesis Candidate Genes in the AZFc Region of the Y Chromosome by Multiplex Real Time PCR Followed by High Resolution Melting Analysis
Source: PLoS One. 2014 May 14;9(5):e97227. doi: 10.1371/journal.pone.0097227 (PMC4020812; doi:10.1371/journal.pone.0097227)
Supplement: Protocol S1 — Detailed SYTO 9 multiplex reaction protocol. (DOC) [file pone.0097227.s002.doc]

**Protocol S2.** Detailed SYTO 9 multiplex reaction protocol

Multiplex PCR reaction, was carried out in 25 μL final volume containing 37.5 pmol SYTO 9 (Life Technologies, USA), 2 units GoTaq DNA Polymerase (Promega, USA), 5X Colorless GoTaq Reaction Buffer (Promega, USA), 20 nmol dNTPs (Promega, USA), primers (Integrated DNA Technologies, USA) (as indicated in Table 1) and 2 μL DNA template.

Master mix was prepared as follows:

| **Reagent** | **Volume (μL)** |
| --- | --- |
| 5X Colorless GoTaq Reaction Buffer | 5 |
| 50 μM SYTO 9 | 0.75 |
| 100 mM 4 dNTPs mix | 0.2 |
| 5 pmol AMELxy primer mix | 1.6 |
| 5 pmol GOLGA2LY primer mix | 1.8 |
| 5 pmol CSGP4LY primer mix | 1.8 |
| 5 pmol BPY2 primer mix | 1.8 |
| 5 pmol CDY primer mix | 2.7 |
| 5 pmol PRY primer mix | 2.7 |
| 5 pmol DAZ primer mix | 3.1 |
| 5 pmol SRY primer mix | 3.125 |
| 500u GoTaq DNA Polymerase | 0.4 |

*Note: F and R primers are stored as primer mix at a concentration of 5 pmol/μL each.*

Reactions were prepared on a RotoCycler 72 Workstation cooled aluminum plate (Corbett Research, Australia) in 0.1 mL tubes. First, 23 μL of master mix and then 2 μL of DNA template (0.3125-10 ng/μL) were pipetted into the tubes. The PCR reaction was performed in a Rotor Gene 6000 Real Time PCR equipment (Corbett Life Science, Australia). PCR cycling conditions were: initial denaturation at 95°C for 1 min followed by 30 cycles with 94 °C 10 s/ 62 °C 30 s/ 72 °C 30 s. Following amplification, a High Resolution Melting (HRM) analysis was performed ranging from 74 °C to 91 °C at 0.2 °C/s temperature increase. Fluorescence detection was performed in the green channel and gain setting were set as follows: detection on the green channel at the end of the extension step (72°C) with auto-gain optimization between 5-10 Fl and HRM gain optimization with highest fluorescence less than 100.
