# Supplementary material for: High-Throughput Screening for Spermatogenesis Candidate Genes in the AZFc Region of the Y Chromosome by Multiplex Real Time PCR Followed by High Resolution Melting Analysis
Source: PLoS One. 2014 May 14;9(5):e97227. doi: 10.1371/journal.pone.0097227 (PMC4020812; doi:10.1371/journal.pone.0097227)
Supplement: Table S1 — Primers used for Y-STS amplification. (DOC) [file pone.0097227.s003.doc]

***Table S3.*** *Primers used for Y-STS amplification*

| ***Primer*** | ***Sequence* (5´-3´)** | ***Volume (µL)*** |
| --- | --- | --- |
| *sY1191 F* | *ACATTTTGCTGCCGGTCACCAGACGTTCTACCCTTTCG* | *0.2500* |
| *sY1191 R* | *GAGCCGAGATCCAGTTACCA* |  |
| *sY1206 F* | *ACATTTTGCTGCCGGTCAATTGATCTCCTTGGTTCCCC* | *0.3125* |
| *sY1206 R* | *GACATGTGTGGCCAATTTGA* |  |
| *sY1291 F* | *ACATTTTGCTGCCGGTCATAAAAGGCAGAACTGCCAGG* | *0.7000* |
| *sY1291 R* | *GGGAGAAAAGTTCTGCAACG* |  |
| *sY1201 F* | *ACATTTTGCTGCCGGTCACCGACTTCCACAATGGCT* | *0.7000* |
| *sY1201 R* | *GGGAGAAAAGTTCTGCAACG* |  |
| *sY1261 F* | *ACATTTTGCTGCCGGTCAAAGGAGCTTGCCTCATACAATG* | *0.5000* |
| *sY1261 R* | *TTAGAGCTTGCAAGAAGAGTCTAGTAC* |  |
| *sY142 F* | *ACATTTTGCTGCCGGTCAAGCTTCTATTCGAGGGCTTC* | *0.1875* |
| *sY142 R* | *CTCTCTGCAATCCCTGACAT* |  |
| *sY1197 F* | *ACATTTTGCTGCCGGTCATCATTTGTGTCCTTCTCTTGGA* | *0.5000* |
| *sY1197 R* | *TCAAGCCAGGAACTTGCCAC* |  |

** All primer volumes are referred to a primer mix with 10:40 pmol Forward/Reverse ratios.*
